# Mr. Duck, on the Case of Eliz. Crocker

**Published:** 1808-09

**Authors:** N. Duck

**Affiliations:** Bristol


### *To the Editors of the Medical and Physical Journal.*

Gentlemen,

With your permission, I beg to offer a few words in reply to T. H's observations on the case of Eliz. Crocker, which appeared in your Journal for June last. The case was drawn up for the perusal, and read at the Annual Meeting of the Western Medical Society, which was numerously attended at Sherborne, in June 1807. It was then verbally explained, that neither Dr. Proctor, nor myself, considered it as a gangrene of the whole annular substance of the intestine, but of a portion of one of the small intestines, probably of the ilium, extending through about half its circumference. It was considered remarkable by several gentlemen present, that five inches of the *side* of the intestine should adhere and heal in such a manner ner as to leave no fistulous opening through which the fæces could find an exit, and that the case was worth recording, to show what nature is sometimes capable of effecting.—It was therefore left with Dr. P. the president, to dispose of as he might think proper, with a request that he would furnish me with a copy of it, as I had not kept one. Had I received the copy before the case appeared in the Journal, I should of course have added this explanation, which would have tended to remove every doubt in the mind of T. H. In this view, Pike's case would not have appeared inapplicable, so far as the general progress and termination of the disorder were concerned. In his case, the caput cæci, with the appendicula vermiformis, were sphacelated; in Crocker's it was presumed (as stated above) that a portion of the ilium, extending through about half its circumference, had been thrown off. Under the present circumstances, however, a great deal must rest upon conjecture, as the portion of sphacelated gut was unfortunately lost; and notwithstanding the attendants upon the woman at the time still assert, that the whole extent of the intestine was annular, the true state of the case cannot be ascertained during the person's existence; but I trust an opportunity may be afforded of doing so after her death. I presume I have now said enough to satisfy T. H. that I did not quote Pike's case with the design he has attributed to me; indeed, it would have been truly extraordinary if I had. With respect to the discharge by stool in Crocker's case, noticed by T. H. it need not, I imagine, excite much wonder, (supposing five inches of the whole circumference of the intestinal canal to be in a state of gangrene) that the fæces should pass through only the day before the separation took place; as this may, I think, be easily reconciled, when we consider there could at least be no resistance in the dead part to the passage of the fæces, (the strangulation being removed) and that the part, *even in that state*, would serve to keep up the continuity of the canal until it might separate.

I am, &c.

N. DUCK.

*Bristol, August 9, 1808.*

